# Evolution of 29 Anthropometric, Nutritional, and Cardiometabolic Parameters Among Morbidly Obese Adolescents 2 Years Post Sleeve Gastrectomy

**DOI:** 10.1007/s11695-017-2868-2

**Published:** 2017-08-18

**Authors:** Wahiba Elhag, Walid El Ansari, Sama Abdulrazzaq, Ali Abdullah, Mohamed Elsherif, Israa Elgenaied

**Affiliations:** 1 Department of Bariatric Surgery, Hamad General Hospital, Hamad Medical Corporation, Doha, State of Qatar; 2 Department of Surgery, Hamad General Hospital, Hamad Medical Corporation, PO Box 3050, Doha, State of Qatar; 3 College of Medicine, Qatar University, Doha, State of Qatar; 4 School of Health and Education, University of Skövde, Skövde, Sweden; 5 Department of Internal Medicine, Hamad General Hospital, Hamad Medical Corporation, Doha, State of Qatar

**Keywords:** Adolescents, Obesity, Sleeve gastrectomy, Bariatric surgery, Nutrient deficiency, Macronutrients, Cardio metabolic risk factors

## Abstract

**Background:**

Laparoscopic sleeve gastrectomy (LSG) is a popular treatment for adolescent morbid obesity. Research on LSG outcomes among adolescents assessed a narrow range of anthropometric, nutritional, or cardiometabolic parameters, leading to an incomplete picture of these changes. We examined a wide variety of anthropometric, nutritional, and cardiometabolic parameters among adolescents before and after LSG.

**Methods:**

We retrospectively reviewed medical charts of all obese adolescents who underwent LSG at Hamad Medical Corporation, Qatar, between January 2011 and June 2015 (*N* = 102). We assessed preoperative levels and postoperative changes in 4 anthropometric, 15 nutritional, and 10 cardiometabolic parameters.

**Results:**

The study sample comprised 79 patients with complete information (36 males, mean age 15.99 ± 1.1 years). At a mean of 24.2 months post-LSG, we observed (1) significantly reduced mean weight and body mass index by 51.82 ± 28.1 kg and 17 ± 6.24 kg/m^2^, respectively; (2) the highest prevalence of post-LSG deficiencies pertained to vitamin D, albumin, and ferritin (89.3, 38, and 33.3%, respectively); (3) low hemoglobin levels (29.3%) only in females; (4) trace elements were not deficient; (4) significant reductions in percentage of adolescents with elevated low-density lipoprotein (from 66.1 to 38.9%), alanine aminotransferase (from 45.3 to 10.9%), and aspartate aminotransferase (from 24.1 to 8.6%) levels; (5) 100% remission of prediabetes cases; and (6) 80% remission of type 2 diabetes cases.

**Conclusions:**

LSG achieved significant weight loss and improvement of cardiometabolic risk factors among adolescents. However, the slight worsening of preexisting nutritional deficiencies warrants careful preoperative surveillance and appropriate postoperative nutritional supplementation.

## Introduction

Adolescent obesity, a serious public health challenge, can persist into adulthood and contribute to premature development of type II diabetes (T2DM), dyslipidemia, increased cardiovascular disease, reduced quality of life, and early mortality [1, 2]. As nonsurgical treatments (e.g., lifestyle modification/s, medication/s) are modestly effective for extremely obese adolescents [3], bariatric surgery has been increasingly utilized to achieve weight loss and resolution of comorbidities among such adolescents.

Laparoscopic sleeve gastrectomy (LSG) is increasingly employed in adolescents [4], with good safety profile, durable weight loss, and comorbidity resolution [5]. Although LSG is primarily a restrictive (rather than malabsorptive) procedure, it carries the risk of some nutritional deficiencies (NDs) related to postoperative restricted caloric intake, food intolerance, and/or insufficient supplementation [6]. Moreover, preoperative nutritional deficiencies (due to high consumption of energy dense and nutritionally poor food), could worsen after LSG, if not recognized and treated [7, 8]. Nevertheless, LSG can improve obesity-related cardiometabolic risk factors [9].

The literature on adolescent LSG reveals several gaps in terms of nutritional deficiencies and cardiometabolic outcomes. First, most bariatric adolescent studies have focused on gastric bypass/gastric band rather than on LSG [10]. Additionally, most post-LSG adolescent research evaluated weight loss and perioperative complications [11, 12], overlooking the postoperative nutritional changes that are critical in adolescence. An exception is a post-LSG study of a narrow range of cardiometabolic and nutritional deficiency outcomes [9] that did not assess any trace elements, despite their importance. Moreover, the evidence regarding post-LSG comorbidity resolution in adolescence is scarce, with many shortcomings, that include the reporting of: (1) only crude outcomes (e.g., dyslipidemia, with no details of individual lipids) [13], or only single lipids (e.g., triglycerides or high-density lipoproteins) [14, 15]; or, (2) only cardiometabolic outcomes with no nutritional deficiency parameters [14]. In addition, despite the rising adolescent obesity rates in the Eastern Mediterranean Region [16], there exits only two published studies on LSG outcomes in adolescents: both examined cardiometabolic outcomes (no nutritional deficiency assessment) [17, 18], where the latter study had short follow-up (8 months).

Therefore, the current study bridged these gaps, comparing mean preoperative values of 4 anthropometric, 15 nutritional, and 10 cardiometabolic outcomes with postoperative changes at 2 years. We also assessed the postoperative evolution of de novo cases of nutritional and cardiometabolic outcomes. These factors highlight the importance of the current study and its contribution to the emerging evidence base.

## Materials and Methods

### Study Design, Ethics, and Participants

This retrospective study comprised all adolescents [*N* = 102, aged 13–17 years, body mass index (BMI) ≥ 40 or ≥ 35 with comorbidities] who underwent primary or revisional LSG between January 2011 and June 2015 at the Bariatric and Metabolic Surgery Centre, Hamad Medical Corporation, Doha, Qatar. The majority of the sample (97.5% of patients) had undergone primary LSG procedures, and only two patients had conversion from laparoscopic gastric band. The study was approved by Hamad Medical Corporation (Protocol No. 16308116). Follow-up information was missing for 23 adolescents; hence, the current analysis included the data of 79 adolescents.

### Procedures and Data Collection

We reviewed patients’ medical charts/electronic records and retrieved data that included demographics and preoperative and 2-year postoperative information on

1. Anthropometric outcomes [weight, BMI, excess weight (EW) reduction, excess weight loss percentage (EWL%)].
2. Nutritional deficiency outcomes [hemoglobin, hematocrit, mean corpusacular volume (MCV), iron, ferritin, total protein, albumin, calcium, magnesium, phosphate, zinc, copper, vitamin D, vitamin B12, folic acid].
3. Cardiometabolic outcomes [changes in total cholesterol (TC), low-density lipoprotein (LDL), high-density lipoprotein (HDL), triglycerides (TGs), alanine aminotransferase (ALT), aspartate aminotransferase (AST), fasting blood glucose (FBG), hemoglobin A1c (HbA1c), parathyroid hormone (PTH), and uric acid levels].

Standard referential values were adopted for all biochemical blood assays.

### Assessment of Weight Change

We assessed BMI [weight (kg) / height (m^2^)], EW [current weight (kg) − ideal body weight (kg)], EWL% [(preoperative weight − current weight / (preoperative weight − ideal body weight) × 100] [19] and ideal body weight (kg) [22 × height (m^2^)] [20].

### Definition of Cardiometabolic Comorbidities

We defined dyslipidemia as TC ≥ 5.17 mmol/L, LDL ≥ 3.36 mmol/L, HDL ≤ 1 mmol/L, and TG ≥ 1.4 mmol/L; type 2 diabetes as FBG ≥ 7 mmol/L or HbA1c ≥ 6.5%); and prediabetes as HbA1c 5.7–6.4% or FBG 5.6–6.9 mmol/L [21, 22].

### Definition of Cardiometabolic Comorbidity Resolution

We focused only on complete resolution of comorbidities. We defined lipid resolution as TC < 5.17 mmol/L, LDL < 2.8 mmol/L, HDL > 1.2 mmol/L, and TG < 1 mmol/L; prediabetes resolution as FBG < 5.5 mmol/L and HbA1c < 5.7%); and T2DM resolution as FBG < 5.5 mmol/L and HbA1c < 6% without antidiabetic medication [21, 23].

### Surgical Technique

Procedure started with division of gastro-splenic ligament along the greater curvature 4 cm from the pylorus up to the left diaphragmatic crus with ultrasonic shears. Stomach was then mobilized and divided along the lesser curvature from antrum (4 cm from pylorus) up to the angle of His using buttressed (SeamGuard) linear 60-mm stapler (Covidien Tristapler) or Echelon Flex over the calibration tube (Midsleeve 38 Fr) introduced into the stomach. Specimen was removed through the umbilical port. Procedure was concluded with methylene blue leak test.

### Nutritional Deficiency Management Protocol

Adolescents with vitamin/s and/or mineral deficiencies in the preoperative assessment received treatment accordingly prior to surgery. In addition, any ND identified postoperatively was addressed as indicated. All patients were restricted to a liquid diet during the first three postoperative weeks with protein shake supplement provided for each patient to ensure adequate protein intake. Standardized nutritional supplements were also provided. These included daily multivitamin tablets containing vitamins A (retinol) C, D3, E K, B1 (thiamine), B2, B3, B5, B6 (pyridoxine), B8, B12 (cobalamin), and folate and the minerals chrome, iron, magnesium, selenium, and zinc.

### Statistical Analysis

Categorical and continuous values were expressed as frequencies (percentages) and means ± SDs. Descriptive statistics were used to summarize demographic, clinical, anthropometric, nutritional, cardiometabolic, and liver function parameters and other related characteristics. Kolmogorov-Smirnov test was used to test for normality of the data. Paired *t* test was used to compare preoperative and postoperative (2 years post-LSG) mean changes in anthropometric, nutritional, cardiometabolic, and liver function parameters (quantitative outcome measures). Statistical analyses were performed using statistical package SPSS 22.0 (SPSS Inc., Chicago, IL); two-tailed *P* values < 0.05 were considered statistically significant.

## Results

### Changes of Anthropometric Parameters

The sample comprised 36 males (45%), mean age 15.99 ± 1.07 years (range, 13–17 years). At 2-year follow-up (*M* = 24.2 ± 12.2 months), significant reductions were found in weight, BMI, and excess weight (Table 1).

**Table 1:** Mean preoperative and postoperative anthropometric values of the study sample

Parameter	Preop *M* (SD)	Postop^a^ *M* (SD)	*P*	Difference *M* (SD)
Weight	126.15 (22.76)	78.5 (13.17)	< 0.0001	51.82 (28.05)
BMI (kg/m^2^)	46.04 (5.99)	28.30 (6.07)	< 0.0001	17 (6.24)
EW (kg)	66 (19.54)	21 (14.9)	< 0.0001	47.94 (19.84)
EWL (%)	–	81.08 (19.65)	–	–

*M* mean, *SD* standard deviation, *Difference* preop value minus postop value, *BMI* body mass index, *EW* excess weight, *EWL (%*) excess weight loss (%), — not applicable

^a^Measured at a mean of 24.2 months

### Changes of Nutritional Parameters

Table 2 shows that the only preoperative deficiency pertained to vitamin D (*M* = 11.73 ± 6.06 ng/mL), and this deficiency persisted postoperatively (*M* = 17.73 ± 9.33 ng/mL). The preoperative and postoperative mean values of all remaining nutritional parameters were normal. Compared to preoperative levels, significant increases in the mean levels of hematocrit (only for males), MCV, iron, and vitamin D were detected postoperatively; conversely, significant decreases were found in mean levels of total protein, copper, and vitamin B12.

**Table 2:** Preoperative and postoperative mean values of nutritional parameters

Parameter	Normal values (unit)	Preop	Postop^a^	Difference *M* ± SD	*P*
		*M* ± SD	*M* ± SD		
Hemoglobin					
Male	13.8–16 (mg/dL)	14.10 ± 1.25	14.43 ± 2.01	0.28 ± 1.9	0.40
Female	12.1–16 (mg/dL)	12.52 ± 1.15	12.29 ± 1.34	0.22 ± 1.1	0.17
Hematocrit					
Male	34.8–43.9 (%)	43.3 ± 2.93	44.79 ± 2.42	1.3 ± 2.8	0.01
Female	34–40.7 (%)	38.73 ± 2.89	38.01 ± 3.68	− 0.74 ± 3.74	0.21
MCV	83–101 (f/L)	81.16 ± 6.03	83.45 ± 6.68	2.29 ± 4.12	< 0.0001
Iron	5.4–28.6 (μmol/L)	10.31 ± 4.87	15.11 ± 7.87	4.80 ± 4.89	< 0.0001
Ferritin	11–304 (mcg/L)	29.50 ± 25.19	34.72 ± 37.79	5.22 ± 25.62	0.64
Total protein	64–83 (g/L)	73 ± 4.16	69.88 ± 4.63	− 3.13 ± 5.36	< 0.0001
Albumin	40–150 (mg/L)	41.14 ± 4.06	40.38 ± 4.67	− 0.77 ± 4.16	0.13
Calcium	2.1–2.5 (mmol/L)	2.28 ± 0.12	2.55 ± 1.79	0.28 ± 1.81	0.32
Magnesium	0.72–1.04 (mmol/L)	0.81 ± 0.04	0.81 ± 0.04	0.00 ± 0.06	0.91
Phosphate	0.89–1.66 (mmol/L)	1.55 ± 0.22	1.50 ± 0.19	− 0.05 ± 0.15	0.40
Zinc	10.1–16.8 (μmol/L)	12.75 ± 1.25	11.98 ± 1.22	− 0.77 ± 1.84	0.28
Copper	11–22 (μmol/L)	21.32 ± 2.26	16.87 ± 3.45	− 4.45 ± 3.91	0.04
Vitamin D	30–50 (ng/mL)	11.73 ± 6.06	17.73 ± 9.33	6.00 ± 11.11	< 0.0001
Vitamin B12	133–675 (pmol/L)	256.51 ± 106.71	271.22 ± 227.64	− 14.71 ± 229.17	0.036
Folic acid	4–45 (nmol/L)	20.41 ± 11.30	14.40 ± 10.74	− 6.01 ± 12.42	0.07

*M* mean, *SD* standard deviation, *Difference* postoperative value minus preoperative value, *MCV* mean cell volume

^a^Mean follow-up = 24.2 ± 12.28 months

Table 3 shows the subset of the sample with preoperative or postoperative nutritional deficiencies or both. Preoperatively, vitamin D deficiency was observed across most (96.4%) adolescents, followed by ferritin, albumin, iron, and other deficiencies (range, 4.7–39.4%). Postoperatively, vitamin D deficiency was still observed in 89.3% of adolescents, followed by albumin, ferritin, hemoglobin, iron, and other deficiencies (range, 4.6–38%). However, the differences between preoperative and postoperative deficiencies were not statistically significant.

**Table 3:** Evolution of nutritional deficiencies among the study sample

Parameter	Preop *N* (%)	Postop^a^ *N* (%)	*P* ^b^	De novo *N* (%)
Hemoglobin				
Male	13 (39.4)	5 (15.2)	0.02	1 (3.0)
Female	13 (31.7)	12 (29.3)	0.99	4 (9.8)
Hematocrit				
Male	0 (0)	2 (5.9)	NA	5 (5.9)
Female	2 (4.9)	4 (9.8)	0.69	4 (9.8)
MCV (micro def.)^d^				
Male	7 (20.6)	2 (5.9)	0.06	0 (0)
Female	11 (27.5)	8 (20)	0.45	2 (5)
Iron	5 (20.8)	4 (16.7)	0.99	1 (4.2)
Ferritin	2 (33.3)	2 (33.3)	0.99	1 (16.7)
Total protein	0 (0)	3 (4.6)	NA	3 (4.6)
Albumin	21 (29.6)	27 (38)	0.29	14 (19.7)
Calcium	2 (4.7)	2 (4.7)	0.99	1 (2.3)
Magnesium	0 (0)	0 (0)	NA	0 (0)
Phosphate	0 (0)	0 (0)	NA	0 (0)
Zinc	0 (0)	0 (0)	NA	0 (0)
Copper	0 (0)	1 (16.7)	NA	1 (16.7)
Vitamin D	54 (96.4)	50 (89.3)	0.29	2 (3.6)
Vitamin B12	3 (6.7)	6 (15.3)	0.38	4 (8.9)
Folic acid	0 (0)	0 (0)	NA	0 (0)

*N* number, *NA* not applicable, *MCV* mean cell volume

^a^Mean follow-up = 24.2 ± 12.28 months

^b^McNemar chi-squared test

^c^Percentage of participants with deficiency calculated as the number of participants (among those for whom sufficient data were available both preoperatively and postoperatively to determine whether the coexisting condition was present), divided by the number of participants (among those for whom sufficient data were available preoperatively and postoperatively)

^d^Representing microcytic deficiency (MCV < 83) in anemic patients only (i.e., patient with deficient hemoglobin levels)

### Evolution of Nutritional Deficiencies (De Novo Cases)

Table 3 depicts postoperative de novo cases with nutritional deficiencies. The most frequent de novo deficiency was low albumin (14 patients), followed by hematocrit, hemoglobin, vitamin B12, vitamin D, and other de novo deficiencies (range, 1–5 patient/s). De novo deficiencies of minerals and trace element were either absent (e.g., magnesium, phosphate, zinc) or rare (e.g., copper, one patient).

### Changes of Cardiometabolic Parameters

Table 4 shows elevated levels of preoperative ALT, FBG, HbA1c, and uric acid. Postoperatively, mean values of all initially elevated cardiometabolic parameters returned to normal, except for uric acid, which remained slightly above the upper normal limit (356 ± 85.52 μmol/L). Additionally, significant decreases (i.e., return to normal) were observed for TC, LDL, HDL, TG, FBG, HbA1c, and liver enzymes’ mean values. Conversely, no significant decreases were detected in the mean values of PTH and uric acid.

**Table 4:** Preoperative and postoperative mean values of cardiometabolic parameters

Parameter	Normal value (unit)	Preop *M* ± SD	Postop^a^ *M* ± SD	Difference *M* ± SD	*P*
TC	< 5.17 (mmol/L)	4.57 ± 0.74	4.28 ± 0.61	0.29 ± 0.78	0.02
LDL	< 3.36 (mmol/L)	2.92 ± 0.78	2.67 ± 0.66	0.26 ± 0.73	0.04
HDL	> 1 (mmol/L)	1.14 ± 0.26	1.29 ± 0.33	− 0.14 ± 0.27	0.003
TG	< 1.7 (mmol/L)	1.13 ± 0.47	0.83 ± 0.41	0.30 ± 0.50	0.001
ALT	9–24 (U/L)	31.03 ± 19.54	17.68 ± 15.67	13.35 ± 19.40	< 0.0001
AST	13–26 (U/L)	23.12 ± 9.79	18.14 ± 8.85	4.98 ± 11.85	0.002
FBG	3.5–5.5 (mmol/L)	5.71 ± 3.14	4.85 ± 2.05	0.86 ± 2.18	0.006
HbA1c	4.8–6 (%)	6.55 ± 2.61	5.48 ± 1.44	1.07 ± 1.59	0.001
PTH	15–65 (pg/mL)	56.58 ± 23.23	52.83 ± 40.54	3.75 ± 42.30	0.77
Uric Acid	150–350 (mmol/L)	355 ± 67.97	356 ± 85.52	− 1.11 ± 38.58	0.93

*M* mean, *SD* standard deviation, *Difference* postoperative value minus preoperative value, *TC* total cholesterol, *LDL* low-density lipoprotein, *HDL* high-density lipoprotein, *TG* total triglycerides, *ALT* alanine amino transferase, *AST* aspartate aminotransferase, *FBG* fasting blood glucose, *HbA1c* glycosylated hemoglobin

^a^Mean follow-up = 24.2 ± 12.28 months

### Evolution of Cardiometabolic Risk Factors (De Novo Cases)

Significant postoperative improvements were found in the percentage of patients with elevated LDL, ALT, and AST. Similar improvements were seen in patients with T2DM and prediabetes (Table 5). We also observed a 100% remission among adolescents with preoperative elevated TG levels and prediabetes. The postoperative remissions of the remaining cardiometabolic parameters ranged between 21.1 and 90%. The numbers of de novo cases were generally low across most cardiometabolic parameters, with the exception of HDL (four de novo low HDL cases), LDL (three de novo elevated LDL cases), and AST (three de novo elevated AST cases).

**Table 5:** Evolution of cardiometabolic parameters of the sample

Parameter	Preop	Postop^a^	*P* ^b^	Remission	De novo
	*N* (%)	*N* (%)		*N* (%)	*N* (%)
TC	10 (20.8)	3 (6.3)	0.06	13 (29.5)	4 (9.1)
LDL	11 (66.1)	7 (38.9)	< 0.0001	8 (21.1)	2 (5.37)
HDL	12 (32.4)	7 (18.9	0.27	37 (100)	0 (0)
TG	4 (10.3)	1 (2.6)	0.38	35 (83.3)	0 (0)
ALT	29 (45.3)	7 (10.9)	< 0.0001	22 (75.9)	0 (0)
AST	14 (24.1)	5 (8.6)	0.03	12 (85.7)	3 (5.2)
Prediabetes	17 (12.6)	4 (5.0)	< 0.0001	17 (100.0)	0 (0)
T2DM	10 (12.6)	6 (7.6)	< 0.0001	8 (80.0)	0 (0)
Uric acid	6 (66.7)	3 (33.3)	0.25	3 (50.0)	0 (0)
PTH	4 (33.3)	2 (16.7)	0.63	3 (75.0)	1 (8.3)

*TC* total cholesterol, *NA* not applicable, *LDL* low-density lipoprotein, *HDL* high-density lipoprotein, *ALT* alanine aminotransferase, *AST* aspartate aminotransferase, *T2DM* type 2 diabetes

^a^Mean follow-up = 24.2 ± 12.28 months

^b^McNemar chi-squared test

^c^Percentage of participants with metabolic abnormality calculated as the number of participants (among those for whom sufficient data were available both preoperatively and postoperatively to determine whether the coexisting condition was present), divided by the number of participants (among those for whom sufficient data were available preoperatively and postoperatively)

## Discussion

Few studies have examined the nutritional and cardiometabolic outcomes of adolescent bariatric surgery beyond 1 year [9, 24]. To the best of our knowledge, this is the first research in the Eastern Mediterranean Region assessing a comprehensive range of LSG outcomes. Two years after LSG, adolescents generally showed impressive improvements in anthropometric (weight, BMI, EWL%, Table 1), nutritional (hematocrit, MCV, iron, vitamin D, Table 2), and cardiometabolic (TC, LDL, HDL, TG, T, ALT, AST, Table 2) outcomes.

### Anthropometric Outcomes

We observed significant weight reduction (mean = 51.82 kg) that exceeded post-LSG weight reductions reported by recent studies (39 kg in USA and 40 kg in France) [9, 14]. Interestingly, this higher weight reduction occurred despite the fact that mean BMI in our study was lower than that reported in previously mentioned studies [9], although the literature suggests that, among adults, more weight reduction is usually achieved in patients with higher preoperative BMI [25]. Such controversy suggests that the relationship between the initial BMI and the magnitude of postoperative weight reduction might not be entirely linear, with other intervening factors potentially influencing/moderating this relationship. Future research should explore these relationships. Likewise, mean decline in BMI and EWL% in our study agreed with or exceeded the values reported elsewhere [9, 26]. The finding that most adolescents in our study achieved and maintained a meaningful weight loss for at least 2 years postoperatively suggests that compared to adults, adolescents treated with bariatric surgery may have a better potential for the reversal of anthropometric parameters [9].

### Nutritional Outcomes

Generally, we observed no preoperative or postoperative deficiencies of trace elements/minerals, except for one de novo case of copper deficiency. Adequate levels of trace elements/minerals are important, given their critical roles in the immune system, cell/neuromuscular functions, wound healing, and bone health. We found no comparable published studies on post-bariatric surgery trace element/mineral changes among adolescents. Nevertheless, among adult populations, others have reported post-bariatric surgery deficiencies in zinc of 7–15% and 0–5% in copper [27].

Vitamin D was the most prevalent deficiency in our study (96.4% preoperatively), and exceeded deficiencies reported by others [28, 29]. Moreover, this deficiency persisted 2 years later (89.3% postoperatively) and remained higher than other reports [9]. Such vitamin D deficiency may be driven by underexposure to ultraviolet radiation, malabsorption, and decreased bioavailability due to enhanced uptake by adipose tissue [30]. Despite of persistent vitamin D deficiency preoperatively and postoperatively, the incidence of hypocalcemia remained low (4.7%). On the other hand, hyperparathyroidism related to vitamin D deficiency was present among only 33.3 and 16.7% of adolescents preoperatively and postoperatively, lower than levels in other studies [6, 31], probably due to the high prevalence of vitamin D among our sample. The persistence of postoperative vitamin D deficiency and secondary hyperparathyroidism highlights the need to assess the optimal vitamin D and calcium supplementation doses necessary to preserve bone health.

With regards to hemoglobin, about one third of our adolescents had low preoperative hemoglobin levels, confirming the high risk of anemia among young populations [32]. Postoperatively, the percentage of patients with low hemoglobin levels significantly decreased among males (from 39.4 to 15.2%), but only modestly reduced among females (from 31.7 to 29.3%), probably due to menstrual loss, suggesting the need for additional iron supplementation among young females. However, our percentage of patients with iron deficiency was twice than that reported among adolescents who underwent gastric banding [33], probably due to the decreased hydrochloric acid production essential for iron absorption after gastric resection, along with the reduced intake of iron-rich food such as red meat [34].

In terms of albumin levels, the 29.6% rate of albumin deficiency was high compared with other studies (3%) [9]. The percentage of our patients with hypoalbuminemia increased to 38.3% postoperatively (14 de novo cases), in contrast to other research that reported no albumin deficiency post-LSG [9]. This albumin deficiency can be explained by LSG’s restrictive nature, and the observed intolerance to protein-rich food postoperatively [35]. High post-LSG protein intake is hence encouraged for adolescents especially with the rapid weight loss during the first year. Protein shake supplements can be provided as and alternative in case of protein intolerance.

### Cardiometabolic Outcomes

Generally, the percentage of patients with cardiometabolic abnormalities significantly decreased postoperatively. Post-LSG lipid profile among adolescents is critical, as there is correlation between adolescent combined dyslipidemia (elevated LDL and TG, low HDL) and atherosclerosis, with subsequent premature coronary artery disease [36]. Hence, understanding the lipid dynamics among post-LSG adolescents is critical. In our study, the majority of adolescents exhibited elevated LDL levels preoperatively, higher than others [37]. However, our LDL levels significantly decreased postoperatively, matching findings observed in Saudi Arabia [17]. Likewise, we detected 83.3% hypertriglyceridemia remission rate, confirming previous findings [14, 17]. Such improvements in hypertriglyceridemia after bariatric surgery can be due to reduced abdominal fat and decreased flux of free fatty acids to the liver, resulting in decreased hepatic secretion of triglyceride-rich lipoproteins [38].

In addition, the percentage of our patients with low HDL exceeded that reported for obese adolescents in the USA [37]. Despite such elevated preoperative levels, we observed a significant drop in the low HDL levels postoperatively with (100%) remission rate, higher than reported in by others [15, 17]. Such increase of HDL is attributed to increased physical activity and was found protective against atherosclerosis mainly through suppressing LDL accumulation, inflammation, and thrombosis [39]. Finally, the incidence of our elevated TC preoperatively is within the range reported by others [17, 40], and while it significantly decreased postoperatively, our remission rate (29.5%) was lower than in other studies (58.3%) [41]. Overall, our observed improvements in lipid profile support the proposal that adolescents may have better potential than adults for the reversal of dyslipidemia [9]. However, randomized controlled trials are needed to demonstrate LSG’s long-term effects on reduction of atherogenic lipids and subsequent cardiovascular disease prevention in adulthood.

In terms of uric acid, in Japan, 20.7% of obese adolescents had hyperuricemia [42], probably linked to metabolic syndrome and insulin resistance [43, 44]. Our incidence of preoperative hyperuricemia decreased from 66.7 to 33.3% postoperatively, possibly due to enhanced insulin sensitivity after LSG [45].

Among obese adolescents, an increased incidence of nonalcoholic fatty liver disease (NAFLD) was reported. Elevated aminotransferases act as surrogate NAFLD markers which predict subsequent diabetes, metabolic syndrome, and cirrhosis [46, 47]. Others have reported elevated ALT among obese boys and girls [48]. Our prevalence of elevated ALT levels significantly decreased postoperatively with 75.9% remission rate. Likewise, obese adolescents also have higher prevalence of elevated AST compared to normal weight adolescents (14.9 vs. 6.6%) [49]. Our preoperative AST prevalence, although higher than others [50], significantly decreased postoperatively. We agree with others where liver enzymes improved 1 year after surgery amounting to 55.6% (post-gastric bypass) and 33.3% (post-gastric band) [51], and with a study in Sweden that reported marked AST and ALT improvements post-gastric bypass [24]. Factors that improve liver enzymes include the beneficial effects of weight loss, better insulin sensitivity, improved dyslipidemia, and reduced inflammatory markers [52]. Hence, early bariatric surgery should be considered for adolescents with NAFLD. Future research should further examine such effects.

Although the 12.6% prevalence of prediabetes in our study was higher than that reported by others [2, 53], we observed 100% remission prediabetes rate which is identical to findings in other studies [9, 17]. However, our preoperative T2DM prevalence was lower than that in Saudi Arabia (23%) [17] and USA (14%) [2]. Although T2DM traditionally affects adults, its incidence has dramatically increased among adolescents [54], probably linked to sedentary lifestyle, obesity, and insulin resistance [55]. Our prevalence of T2DM significantly decreased post-LSG (80% remission rate), comparable to other remissions reported after LSG (88.5–100%) [9, 17], gastric banding (59.1%), and gastric bypass (78.6%) [52].

Prediabetes and T2DM remission and concomitant HbA1c improvements are related to better insulin sensitivity and glucose metabolism that influence weight-dependent (body weight loss) and independent (hormonal glucagon-like peptide 1) pathways. This hormone stimulates glucose-dependent insulin secretion from β cells, inhibits glucagon secretion, and may increase peripheral glucose uptake and insulin sensitivity [56]. Given the potential complications of adolescent T2DM, improvements of T2DM post-bariatric surgery provide further evidence for its validity as an effective treatment option of adolescent T2DM [57].

This study has limitations. It would have been beneficial to have patient data on preoperative and postoperative food consumption, diet regimens, and intake of vitamins/other supplements that could have influenced the observed nutritional and cardiometabolic changes. In addition, we provide our post-LSG patients with specific dietary/supplementation protocols, but compliance is difficult to assess. Longer (5 years) post-LSG follow-up of our adolescents’ nutritional profiles would have been useful to assess other longer term effects. Finally, we had a 77.5% follow-up rate, slightly lower than the ideal follow-up of ≥ 80%, which is rarely accomplished even in the most cited bariatric surgery outcome research [58–60]. Nevertheless, the study has several strengths. We examined a wide variety of parameters (25 anthropometric, nutritional, and cardiometabolic). To date, no study has examined, in similar detail, such as broad range of parameters after LSG among adolescents. We focused on only one bariatric procedure (LSG) rather than a range of bariatric techniques (gastric band, gastric bypass); hence, our findings are specific to post-LSG consequences, “unaffected” by results of other bariatric procedures that could have potentially influenced the nutritional/cardiometabolic outcomes after surgery. With a few exceptions, very few studies have achieved such results [9, 17].

## Conclusion

Our findings confirm that, among obese adolescents, LSG achieves significant weight loss and improves anthropometric parameters and 10 cardiometabolic risk factors, without the development of trace element deficiency after surgery. Conversely, the preoperative nutritional deficiencies (vitamin D, anemia, and hypoalbuminemia) persisted or worsened postoperatively. Such nutritional deficiencies warrant careful and specific preoperative surveillance and appropriate postoperative monitoring and nutritional supplementation, especially during the adolescence period, in order to ensure healthy development and growth. Longer follow-up of adolescents’ nutritional profiles is recommended to assess other potential effects of LSG.

## References

[1] Ng M, Fleming T, Robinson M (2014). Global, regional, and national prevalence of overweight and obesity in children and adults during 1980-2013: a systematic analysis for the Global Burden of Disease Study 2013. Lancet Lond Engl.

[2] Michalsky MP, Inge TH, Simmons M (2015). Cardiovascular risk factors in severely obese adolescents: the Teen Longitudinal Assessment of Bariatric Surgery (Teen-LABS) study. JAMA Pediatr.

[3] Barlow SE (2007). Expert committee recommendations regarding the prevention, assessment, and treatment of child and adolescent overweight and obesity: summary report. Pediatrics.

[4] Pallati P, Buettner S, Simorov A (2012). Trends in adolescent bariatric surgery evaluated by UHC database collection. Surg Endosc.

[5] Inge TH, Zeller MH, Jenkins TM (2014). Perioperative outcomes of adolescents undergoing bariatric surgery: the Teen-Longitudinal Assessment of Bariatric Surgery (Teen-LABS) study. JAMA Pediatr.

[6] Aarts EO, Janssen J, Janssen IMC (2013). Preoperative fasting plasma C-peptide level may help to predict diabetes outcome after gastric bypass surgery. Obes Surg.

[7] Kant AK (2003). Reported consumption of low-nutrient-density foods by American children and adolescents: nutritional and health correlates, NHANES III, 1988 to 1994. Arch Pediatr Adolesc Med.

[8] Damms-Machado A, Friedrich A, Kramer KM (2012). Pre- and postoperative nutritional deficiencies in obese patients undergoing laparoscopic sleeve gastrectomy. Obes Surg.

[9] Inge TH, Courcoulas AP, Jenkins TM (2016). Weight loss and health status 3 years after bariatric surgery in adolescents. N Engl J Med.

[10] Paulus GF, de Vaan LEG, Verdam FJ (2015). Bariatric surgery in morbidly obese adolescents: a systematic review and meta-analysis. Obes Surg.

[11] Ejaz A, Patel P, Gonzalez-Heredia R (2017). Laparoscopic sleeve gastrectomy as first-line surgical treatment for morbid obesity among adolescents. J Pediatr Surg.

[12] Speer AL, Parekh J, Qureshi FG (2017). Thirty-day outcomes for children and adolescents undergoing laparoscopic sleeve gastrectomy at a free-standing children’s hospital. Clin Obes.

[13] Boza C, Viscido G, Salinas J (2012). Laparoscopic sleeve gastrectomy in obese adolescents: results in 51 patients. Surg Obes Relat Dis Off J Am Soc Bariatr Surg.

[14] Pourcher G, De Filippo G, Ferretti S (2015). Short-term results of single-port sleeve gastrectomy in adolescents with severe obesity. Surg Obes Relat Dis Off J Am Soc Bariatr Surg..

[15] Davidson WS, Inge TH, Sexmith H (2017). Weight loss surgery in adolescents corrects high-density lipoprotein subspecies and their function. Int J Obes.

[16] ALNohair S. (2014). Obesity in gulf countries. Int J Health Sci.

[17] Alqahtani AR, Elahmedi MO, Qahtani A Al. Co-morbidity resolution in morbidly obese children and adolescents undergoing sleeve gastrectomy. Surg Obes Relat Dis Off J Am Soc Bariatr Surg. 2014;10:842–850. 10.1016/j.soard.2014.01.020 25439000

[18] Kuwari M Al, Gagner M, Sargsyan D, et al. The outcomes of sleeve gastrectomy for treatment of morbidly obese adolescents in Hamad General Hospital. Qatar Found. Annu Res Forum Proc. 2012;BMP131.

[19] Hatoum IJ, Kaplan LM (2013). Advantages of percent weight loss as a method of reporting weight loss after Roux-en-Y gastric bypass. Obes Silver Spring Md..

[20] Lemmens HJM, Brodsky JB, Bernstein DP (2005). Estimating ideal body weight—a new formula. Obes Surg.

[21] Expert Panel on Integrated Guidelines for Cardiovascular Health and Risk Reduction in Children and Adolescents: Summary Report. Pediatrics. 2011;128:S213–56. 10.1542/peds.2009-2107C PMC4536582 22084329

[22] Association AD (2015). 2. Classification and diagnosis of diabetes. Diabetes Care.

[23] Standardized Outcomes Reporting in Metabolic and Bariatric Surgery [Internet]. Am Soc Metab. Bariatr Surg. [cited 2017 May 31] Available from: https://asmbs.org/resources/standardized-outcomes-reporting-in-metabolic-and-bariatric-surgery

[24] Olbers T, Gronowitz E, Werling M (2012). Two-year outcome of laparoscopic Roux-en-Y gastric bypass in adolescents with severe obesity: results from a Swedish Nationwide Study (AMOS). Int J Obes.

[25] Ochner CN, Jochner MCE, Caruso EA (2013). Effect of preoperative body mass index on weight loss after obesity surgery. Surg Obes Relat Dis Off J Am Soc Bariatr Surg.

[26] Alqahtani A, Alamri H, Elahmedi M (2012). Laparoscopic sleeve gastrectomy in adult and pediatric obese patients: a comparative study. Surg Endosc.

[27] Papamargaritis D, Aasheim ET, Sampson B (2015). Copper, selenium and zinc levels after bariatric surgery in patients recommended to take multivitamin-mineral supplementation. J Trace Elem Med Biol Organ Soc. Miner Trace Elem GMS.

[28] Lenders CM, Feldman HA, Scheven EV (2009). Relation of body fat indexes to vitamin D status and deficiency among obese adolescents. Am J Clin Nutr.

[29] Smotkin-Tangorra M, Purushothaman R, Gupta A (2011). Prevalence of vitamin D insufficiency in obese children and adolescents. J Pediatr Endocrinol Metab.

[30] Drincic AT, Armas LAG, Van Diest EE (2012). Volumetric dilution, rather than sequestration best explains the low vitamin D status of obesity. Obes Silver Spring Md.

[31] Inge TH, Jenkins TM, Xanthakos SA (2017). Long-term outcomes of bariatric surgery in adolescents with severe obesity (FABS-5+): a prospective follow-up analysis. Lancet Diabetes Endocrinol.

[32] Bagni UV, Luiz RR, da Veiga GV (2013). Overweight is associated with low hemoglobin levels in adolescent girls. Obes Res Clin Pract.

[33] Nadler EP, Youn HA, Ren CJ (2008). An update on 73 US obese pediatric patients treated with laparoscopic adjustable gastric banding: comorbidity resolution and compliance data. J Pediatr Surg.

[34] Ruz M, Carrasco F, Rojas P (2009). Iron absorption and iron status are reduced after roux-en-Y gastric bypass. Am J Clin Nutr.

[35] Moize V, Geliebter A, Gluck ME (2003). Obese patients have inadequate protein intake related to protein intolerance up to 1 year following roux-en-Y gastric bypass. Obes Surg.

[36] Bibbins-Domingo K, Coxson P, Pletcher MJ (2007). Adolescent overweight and future adult coronary heart disease. N Engl J Med.

[37] May AL, Kuklina EV, Yoon PW (2012). Prevalence of cardiovascular disease risk factors among US adolescents, 1999−2008. Pediatrics.

[38] Hüttl TP, Lang R, To VT (2012). Changes in body weight, glucose homeostasis, lipid profiles, and metabolic syndrome after restrictive bariatric surgery. Exp Clin Endocrinol Diabetes.

[39] Badimon L, Vilahur G (2012). LDL-cholesterol versus HDL-cholesterol in the atherosclerotic plaque: inflammatory resolution versus thrombotic chaos. Ann N Y Acad Sci.

[40] Friedland O, Nemet D, Gorodnitsky N (2002). Obesity and lipid profiles in children and adolescents. J Pediatr Endocrinol Metab JPEM.

[41] Lawson ML, Kirk S, Mitchell T (2006). One-year outcomes of Roux-en-Y gastric bypass for morbidly obese adolescents: a multicenter study from the Pediatric Bariatric Study Group. J Pediatr Surg.

[42] Tang L, Kubota M, Nagai A (2010). Hyperuricemia in obese children and adolescents: the relationship with metabolic syndrome. Pediatr Rep.

[43] Ogura T, Matsuura K, Matsumoto Y (2004). Recent trends of hyperuricemia and obesity in Japanese male adolescents, 1991 through 2002. Metab - Clin Exp.

[44] Yoo TW, Sung KC, Shin HS (2005). Relationship between serum uric acid concentration and insulin resistance and metabolic syndrome. Circ J.

[45] Oberbach A, Neuhaus J, Inge T (2014). Bariatric surgery in severely obese adolescents improves major comorbidities including hyperuricemia. Metabolism.

[46] Jimenez-Rivera C, Hadjiyannakis S, Davila J (2017). Prevalence and risk factors for non-alcoholic fatty liver in children and youth with obesity. BMC Pediatr.

[47] Feldstein AE, Charatcharoenwitthaya P, Treeprasertsuk S (2009). The natural history of nonalcoholic fatty liver disease in children: a follow-up study for up to 20-years. Gut.

[48] Di Bonito P, Sanguigno E, Di Fraia T (2009). Association of elevated serum alanine aminotransferase with metabolic factors in obese children: sex-related analysis. Metabolism.

[49] Kelishadi R, Cook SR, Adibi A (2009). Association of the components of the metabolic syndrome with non-alcoholic fatty liver disease among normal-weight, overweight and obese children and adolescents. Diabetol Metab Syndr.

[50] Corey KE, Stanley TL, Misdraji J (2014). Prevalence and outcome of nonalcoholic fatty liver disease in adolescents and young adults undergoing weight loss surgery. Pediatr Obes.

[51] Messiah SE, Lopez-Mitnik G, Winegar D (2013). Changes in weight and comorbidities among adolescents undergoing bariatric surgery: 1-year results from the bariatric outcomes longitudinal database. Surg Obes Relat Dis Off J Am Soc Bariatr Surg..

[52] Hafeez S, Ahmed MH. Bariatric surgery as potential treatment for nonalcoholic fatty liver disease: a future treatment by choice or by chance? J Obes. [Internet]. 2013 [cited 2017 May 12];2013 Available from: http://www.ncbi.nlm.nih.gov/pmc/articles/PMC3569911/10.1155/2013/839275 PMC3569911 23431426

[53] Cambuli VM, Incani M, Pilia S, Congiu T, Cavallo MG, Cossu E (2009). Oral glucose tolerance test in Italian overweight/obese children and adolescents results in a very high prevalence of impaired fasting glycaemia, but not of diabetes. Diabetes Metab Res Rev.

[54] Dabelea D, Mayer-Davis EJ, Saydah S (2014). Prevalence of type 1 and type 2 diabetes among children and adolescents from 2001 to 2009. JAMA.

[55] Marcovecchio M, Mohn A, Chiarelli F (2005). Type 2 diabetes mellitus in children and adolescents. J Endocrinol Investig.

[56] Nandagopal R, Brown RJ, Rother KI (2010). Resolution of type 2 diabetes following bariatric surgery: implications for adults and adolescents. Diabetes Technol Ther.

[57] Beamish AJ, D’Alessio DA, Inge TH (2015). Controversial issues: when the drugs don’t work, can surgery provide a different outcome for diabetic adolescents?. Surg Obes Relat Dis.

[58] Fewtrell MS, Kennedy K, Singhal A (2008). How much loss to follow-up is acceptable in long-term randomised trials and prospective studies?. Arch Dis Child.

[59] Sjöström L, Narbro K, Sjöström CD (2007). Effects of bariatric surgery on mortality in Swedish obese subjects. N Engl J Med.

[60] Puzziferri N, Roshek TB, Mayo HG (2014). Long-term follow-up after bariatric surgery: a systematic review. JAMA.

